# Infrared Photodetection from 2D/3D van der Waals Heterostructures

**DOI:** 10.3390/nano13071169

**Published:** 2023-03-24

**Authors:** Qianying Tang, Fang Zhong, Qing Li, Jialu Weng, Junzhe Li, Hangyu Lu, Haitao Wu, Shuning Liu, Jiacheng Wang, Ke Deng, Yunlong Xiao, Zhen Wang, Ting He

**Affiliations:** 1 Hangzhou Institute for Advanced Study, University of Chinese Academy of Sciences, Hangzhou 310024, China; 2 University of Chinese Academy of Sciences, Beijing 100049, China; 3 State Key Laboratory of Infrared Physics, Shanghai Institute of Technical Physics, Chinese Academy of Sciences, Shanghai 200083, China

**Keywords:** infrared detection, 2D materials, bulk infrared materials, 2D/3D infrared detectors, heterojunction

## Abstract

An infrared photodetector is a critical component that detects, identifies, and tracks complex targets in a detection system. Infrared photodetectors based on 3D bulk materials are widely applied in national defense, military, communications, and astronomy fields. The complex application environment requires higher performance and multi-dimensional capability. The emergence of 2D materials has brought new possibilities to develop next-generation infrared detectors. However, the inherent thickness limitations and the immature preparation of 2D materials still lead to low quantum efficiency and slow response speeds. This review summarizes 2D/3D hybrid van der Waals heterojunctions for infrared photodetection. First, the physical properties of 2D and 3D materials related to detection capability, including thickness, band gap, absorption band, quantum efficiency, and carrier mobility, are summarized. Then, the primary research progress of 2D/3D infrared detectors is reviewed from performance improvement (broadband, high-responsivity, fast response) and new functional devices (two-color detectors, polarization detectors). Importantly, combining low-doped 3D and flexible 2D materials can effectively improve the responsivity and detection speed due to a significant depletion region width. Furthermore, combining the anisotropic 2D lattice structure and high absorbance of 3D materials provides a new strategy in high-performance polarization detectors. This paper offers prospects for developing 2D/3D high-performance infrared detection technology.

## 1. Introduction

IR light is one type of special invisible radiation whose wavelength is above 760 nm and below 1000 μm. It was discovered by Herschel using thermometers in 1800 [1]. Furthermore, after 1940, the year of the infrared photodetector’s invention based on the photoelectric effect, people began to know its importance in military, remote sensing, communication, and other relevant fields close to people’s lives [2]. In the development of IR photodetectors, new materials have always been an efficient and rewarding way to fabricate better-performance devices. Thin film materials, such as HgCdTe, InSb, and some lead salt compounds, have intrinsic narrow bandgaps [3]. With the improvement of material growth technology, some new quantum materials were created by band engineering, represented by type-Ⅱ superlattice and quantum well or quantum dot material. They all have superior IR photoelectric conversion abilities through which one can obtain high-enough quantum efficiency and response. However, because dark current and assisted noise are very sensitive to temperature, most IR photodetectors must operate at a low temperature to improve their signal to noise ratios [4]. This is understandable, for the energy of circumstance temperature is comparable to a narrow band gap, inducing abundant free carriers and high generation–recombination rates. Therefore, cooler equipment becomes necessary but conflicts with the current IR technology’s miniaturization demand. Moreover, minimizing pixel size can improve image resolution and save the volume and weight of the optical systems, so micro-nano devices and processing technology become another ambition of current IR technology. Dramatically, the emergence of 2D material provides an optional easily integrated candidate for high performance IR photodetectors without coolers [5].

Two-dimensional materials now comprise a well-known new type of semiconductor material [6,7]. Compared with the history of modern infrared (IR) detection technology, 2D materials are very young. On the macro level, 2D materials have an ultra-thin scale [8,9], whereas the thickness is usually between several angstroms (monolayer) and dozens of nanometers [10]. They have a huge surface-to-volume ratio, effectively reducing interlayer transport time [11]. From a micro perspective, they have atomic layers combined by relatively weak van der Waals forces rather than chemical bonds [12], so layered controllable 2D material can be easily obtained by mechanical exfoliation [13]. However, the bond is like a covalent semiconductor within the atomic layers [14], forming a definite but easily regulated band gap [15]. Compared with traditional infrared material, 2D devices avoid surface leakage current benefiting from a few dangling bonds between layers [16].

**Figure 1:**
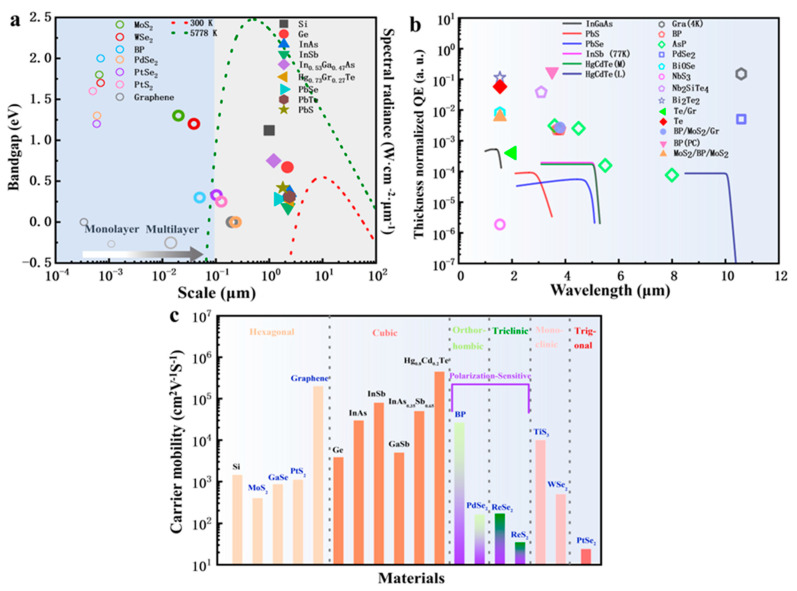
Physical properties comparison of 2D materials and 3D materials. (**a**) Bandgap comparison of 2D materials and 3D materials and spectral radiance for different temperature sources [17,54,55,56,57,58,59,60]. (**b**) Thickness-normalized EQE for different materials: the scatter represents the 2D-layered materials, while the line represents traditional thin film materials [17,45,61,62,63,64,65,66,67,68,69,70,71]. (Hollow symbol represents measured results of laser source, and solid symbol and solid lines represent the result of blackbody source.) (**c**) Comparison of carrier mobility in different crystal system. Graphene and HgCdTe exhibit ultra-high carrier mobility, orthorhombic BP, PdSe_2_ and triclinic ReSe_2_, ReS_2_ exhibit polarization-sensitive properties [57,72,73,74,75,76,77,78,79,80].

As shown in Figure 1b, a comparison of thickness-normalized QE is performed between 2D materials and traditional thin film materials (thickness is normalized at 1 nm). It seems that most 2D layered materials have better photoelectric conversion capability. In the infrared range between 1 and 12 µm, graphene, Bi_2_O_2_Se, BP, AsP, Nb_2_SiTe_4_, and PdSe_2_ have obvious advantages over thin film narrow bandgap material represented by HgCdTe.

Quantum efficiency means the capability of photoelectric conversion. It is always divided into external quantum efficiency (EQE) and internal quantum efficiency (IQE) [16]. EQE is the ratio of collected carriers by IR photodetectors to incident photons. IQE is the ratio of collected electron–hole pairs to the absorbed photons by IR photodetectors. If we suppose that gain and carrier transfer efficiency is equal to 1, then the IQE and EQE will be below 1, but IQE is higher than EQE at some wavelength. The equation of EQE and its relationship to IQE are as follows [17]:

(1) $$\eta_{e}=\frac{I_{ph}}{P_{in}}\cdot \frac{h\nu }{e}=\mathrm{Gain}\cdot \left(1-r\right)\cdot \beta \left(1-e^{-\alpha l}\right)=\mathrm{Gain}\cdot \left(1-r\right)\cdot \eta_{i}$$

(2) $$\;\eta_{i}=\beta \left(1-e^{-\alpha l}\right)$$

where $\eta_{e}$ and $\eta_{i}$ mean the EQE and IQE, respectively; $I_{ph}$ is the net photocurrent; $P_{in}$ is the incident optical power; r is the reflectivity; α is the absorption coefficient; β is carrier transfer efficacy; l is the thickness of the absorption layer of photodetectors; and other symbols have their usual physical meanings. Equations (1) and (2) show that quantum efficiency has a very close relationship to thickness. For 2D materials, thickness is one typical characteristic that will be a key influencing factor for quantum efficiency. Furthermore, the absorption coefficient will be less than 10% even if the thickness achieves 100 nm, which is a non-negligible weakness for IR photodetectors. The IQE will greatly rely on the thickness of nanoscale material. So far, there is no rigorous report stating that 2D material IR photodetectors can achieve a high EQE of over 80% without gain. This will be challenging but significant work [18]. However, the thickness of 2D material is so thin (near 2–3 orders of magnitude lower than IR wavelength) that the internal quantum efficiency (IQE) of a 2D material IR detector can hardly reach a high level [19]. 

As shown in Figure 1a, according to Planck’s law, all objects above absolute zero continually emit all-optical wavelength radiation [20], and the wavelength distribution depends on the temperature of the object [21]. Conventionally, we regard ~300 K as room temperature [22], and our photodetectors or detection systems are surrounded by this 300 K background [23]. The wavelength distribution of background radiation mainly lies in the infrared range [17]. Therefore, even ideal IR photodetectors still have performance limitations influenced by the background. Two-dimensional material IR photodetectors are not only affected by the background but are also limited by their own conditions [24]. Furthermore, the immaturity of material growth and device fabricating processes create many noise sources. In Figure 1a, we can also observe that as the number of layers increases, the band gap of 2D materials gradually decreases. Inversely, the bandgap of 3D materials is relatively stable and can meet the infrared radiation at 300 K–5772 K. However, for 2D materials, only when they reach a certain thickness and have strong absorption can they have a good infrared response. 

Carrier mobility is another important factor affecting material properties, and it is greatly dependent on the quality of 2D material and the design of the device [25]. The traps, band offset, and contact will decrease the carrier transfer efficacy. As shown in Figure 1c, the carrier mobility of graphene has a great advantage, but it is still not as good as HgCdTe. The carrier mobility of other 2D materials is still inferior to that of 3D materials. In addition, the immaturity of material growth and device fabricating processes creates many noise sources. Thus, the inherent limitations of 2D materials make them struggle forward on high-performance infrared photodetectors [17].

Infrared detectors based on 3D traditional bulk materials, such as Si, HgCdTe, InGaAs, etc., have been widely used in military, aviation, communication, and other fields [26,27]. Unlike 2D material, the optical absorption thickness of 3D materials can reach 1 µm or more [28], which is closer to the waveband of infrared radiation and more conducive to the absorption [29]. Among them, silicon photonics is compatible with the complementary metal-oxide-semiconductor (CMOS) process, which helps to realize the large-scale integrated development of infrared detectors [30]. However, even so, developing high-performance infrared photodetectors based on 3D materials still faces challenges [31]. Importantly, material quality determines the performance of photodetectors. Conventional preparation of heterostructures requires epitaxial growth techniques, such as molecular beam epitaxy (MBE) and metal–organic chemical vapor deposition (MOCVD) [32,33]. The costly and demanding epitaxy technologies provide high-quality multilayer heterogeneous structures for various complex devices [34,35]. However, they rely on the chemical bonds on the surface and require the epitaxy of materials with similar lattice constants and structures [36]. It is difficult for epitaxy materials with different lattice structures to grow together [37,38]. Furthermore, defects such as dislocations caused by lattice mismatches at heteroepitaxial interfaces increase the generation and recombination current in the space charge region [39]. Generally, a buffer layer is added during growth to reduce dislocation defects, but dislocation due to lattice mismatches is not fully resolved [40,41]. These problems will affect detector performance, leading to large dark current, low quantum efficiency, and low response speed [42]. 

**Figure 2:**
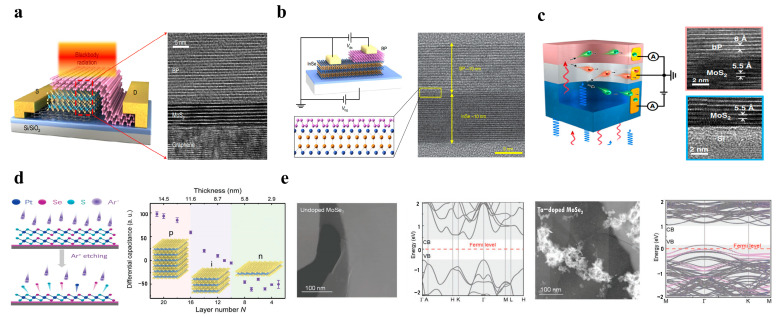
High-quality van der Waals heterojunction interface and modulation of 2D materials P, N conduction type. Schematic diagrams and TEM images of (**a**) BP/MoS_2_/graphene photodetector [45] (copyright 2021, Springer Nature). (**b**) InSe/BP heterojunction [46] (copyright 2019, Springer Nature). (**c**) BP/MoS_2_/Si two-color infrared detector [48] (copyright 2022, Springer Nature). (**d**) Schematic diagram of conductive type of PtSSe with controllable layer thickness switching by Ar+ plasma etching [52] (copyright 2021, John Wiley and Sons). (**e**) Schematic diagram of Ta doping to achieve changing of MoSe_2_ conduction type [53] (copyright 2021, John Wiley and Sons).

Van der Waals integration provides a new method for preparing complex heterostructures. Materials can be physically assembled through van der Waals force interactions [38,43]. This physical assembly method does not rely on one-to-one chemical bonds and requires similar lattice structures [38,44]. Clear transmission electron micrograph (TEM) images of BP/MoS_2_/graphene unipolar barrier photodetectors [45] and InSe/BP avalanche photodetectors [46] have demonstrated that high-quality 2D devices have atomically clear junction interfaces after the dry transfer, as shown in Figure 2a,b. In principle, this van der Waals integration does not require lattice matching and is not limited by material dimensions [47]. Thus, designing and integrating various van der Waals heterostructures with different material combinations become realistic [44]. In Figure 2c, the TEM images of the BP/MoS_2_/Si two-color infrared detector show the existence of high-quality and clean 2D/3D van der Waals heterojunctions [48]. These properties give 2D/3D heterostructure good application prospects in multi-dimensional infrared photodetection [49].

Furthermore, 2D materials can flexibly adjust carrier transmission and switch conduction types by substituting doping, changing layer thickness, field modulation, and chemical treatment [50,51]. This provides excellent flexibility in the manufacture of devices such as photodetectors. As shown in Figure 2d, Wang et al. achieved the transformation of N-type, intrinsic, and P-type conductance by changing the thickness of PtSSe and WSe_2_ [52]. As seen in Figure 2e, with the increase of Ta doping concentration, the conduction type of MoSe_2_ changes from N-type to P-type. A high-performance homojunction photodetector composed of N-type and P-type MoSe_2_ are produced with low power consumption [53]. 

**Figure 3:**
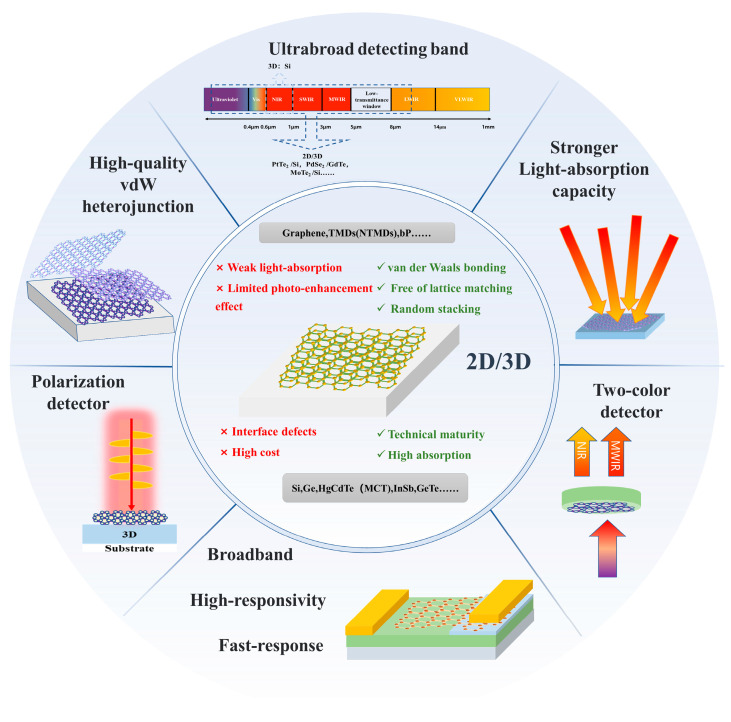
Advantages and disadvantages of 2D and 3D materials and the superiority of 2D/3D infrared photodetection.

Advantages and disadvantages of 2D materials and 3D materials are respectively listed in the inner circle of Figure 3. Two-dimensional materials have weak light-absorption abilities and limited light enhancement responses, but they have van der Waals forces, are free of lattice matching and random stacking, and can be flexibly prepared. Three-dimensional materials have surface defects and are expensive, but they have strong light-absorption abilities and mature technology. As shown in the upper part of Figure 3, the 2D/3D hybrid van der Waals heterojunction has the advantages of a high-quality van der Waals heterojunction, ultrabroad detecting band, and stronger light-absorption capacity. As shown in the lower part of Figure 3, these advantages help to improve the performance of infrared detectors (broadband, high responsivity, fast response) and develop new functional devices (two-color detector and polarization detector).

In this review, 2D/3D infrared detectors with high-quality complex heterostructures realize high-performance and multi-functional infrared detectors. At first, we systematically summarize the material physical properties of 2D and 3D materials related to detection capability, including thickness, band gap, absorption band, quantum efficiency, and carrier mobility. Then, the primary research progresses of 2D/3D infrared detectors in recent years are reviewed from two aspects of performance improvement (broadband, high responsivity, fast response) and new functional devices (two-color detectors, polarization detectors). Finally, we propose the challenges and perspectives of 2D/3D detectors to outline future research directions. 

## 2. Performance Improvement

### 2.1. Broadband

Two-dimensional materials have been demonstrated to have ultra-wide wavelength detection capability from ultraviolet to terahertz [42], and the bandgap of 2D materials is tunable with the number of layers. However, infrared detectors based on 2D materials have large dark currents and low responsivity due to their low light-absorption rate and lack of high-quality junctions [81]. The range of wavelengths that can be responded to is also limited [82]. Compared with 2D materials, 3D materials, such as Si, InGaAs, etc., are suitable for near-infrared photoelectric detection, and HgCdTe is suitable for medium and long-wave infrared detection [16]. The absorption spectrum bands of various 3D and 2D materials are different. According to the design requirements of different bands, the detection of a certain band can be achieved through material selection and the use of various physical mechanisms. The 2D/3D system is expected to realize broadband detection from deep ultraviolet to terahertz.

**Figure 4:**
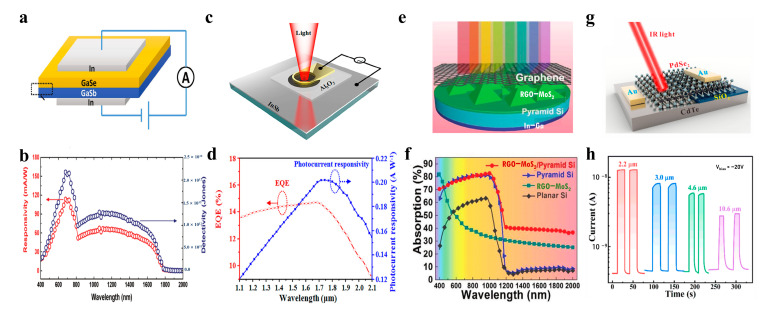
Broadband infrared photodetection. (**a**) Schematic diagram of p-GaSe/n-GaSb vertical heterostructure detector [83] (copyright 2017, John Wiley and Sons). (**b**) Wavelength-dependent photoresponsivity of GaSe/GaSb detector [83] (copyright 2017, John Wiley and Sons). (**c**) Schematic diagram of ultra-broadband Gr/InSb heterostructure photodetector [84] (copyright 2017, AIP Publishing). (**d**) Current–voltage characterizations of Gr/InSb heterostructure photodetector (473 nm–10 μm) [84] (copyright 2017, AIP Publishing). (**e**) Schematic diagram of RGO–MoS_2_/pyramid Si detector [85] (copyright 2018, John Wiley and Sons). (**f**) UV–NIR absorption spectra of RGO–MoS_2_/pyramid Si detector [85] (copyright 2018, John Wiley and Sons). (**g**) Schematic diagram of PdSe_2_/CdTe heterojunction infrared detector for broadband to 10.6 µm [82] (copyright 2021, American Chemical Society). (**h**) Photoresponse properties of PdSe_2_/CdTe detector [82] (copyright 2021, American Chemical Society).

For the different detection ranges of 2D materials and 3D materials, a hybrid dimensional van der Waals heterojunction was built to achieve wavelength expansion. In 2017, as shown in Figure 4a, Peng Wang et al. used molecular epitaxial growth to deposit p-type 2D material GaSe on an n-type substrate GaSb, forming a vertical van der Waals heterostructure [83]. The device can realize a wide-spectrum detection of 400–1800 nm. The depletion region in GaSe extends to 63% at a bias voltage of −1V, taking the responsibility of light absorption and photoexcited carrier generation. At the same time, in the depletion region at the heterojunction, a strong built-in electric field is generated to realize the rapid separation of carriers. Moreover, the ultra-thin vertical distance between GaSe and electrodes reduces carrier recombination and improves the response speed. From the continuous spectral response of the device in Figure 4b, the corresponding responsivity of peaks at 680 nm and 1060 nm are 115 mA/W and 68 mA/W, respectively [83]. The band gaps of both GaSb and GaSe are smaller than the energy of visible light, resulting in a strong response in the visible waveband. However, the infrared response is mainly attributed to GaSb. Because the depletion region of GaSb is much wider than that of GaSe, the response is almost equivalent to infrared and visible light.

Graphene has an absorption spectrum from ultraviolet to far infrared and has high carrier mobility and environmental stability. These characteristics make it possible to use graphene to prepare broadband infrared detectors. In 2017, Jianbao Xu et al. developed a p-type graphene/n-type InSb heterostructure infrared detector achieving ultra-broadband detection from visible light to far infrared at room temperature [84]. They constructed a narrow band gap heterojunction to overcome the low absorption rate of graphene caused by a zero bandgap. InSb with a narrow band gap was used as the substrate, forming a Schottky contact with graphene. In addition, to suppress the dark current, a thin oxide layer of Al_2_O_3_ was introduced between graphene and InSb to create a P–I–N heterojunction, as shown in Figure 4c. Figure 4d shows the photocurrent response of the device in the whole band from 473 nm to 10 μm. In the range of 300–600 nm, both graphene and InSb contribute to the photocurrent, and the response over 600 nm mainly comes from the light absorption of InSb. 

In addition, Peng Xiao et al. prepared an RGO-MoS_2_/pyramid Si heterojunction broadband infrared detector, as shown in Figure 4e [85]. To make up for the limitations of MoS_2_ after thermal decomposition, such as low mobility and defects, they combined MoS_2_ with reduced graphene oxide (RGO) to improve the carrier mobility of MoS_2_ and enhance charge separation/transport. The high-quality heterojunction formed by covering the RGO-MoS_2_ composite film on the pyramid Si has a high light absorption rate. At the same time, the heterojunction can respond to ultraviolet to mid-infrared due to the widened bandgap of thermally decomposed MoS_2_. As shown in Figure 4f, when the wavelength is greater than 1100 nm, the response mainly comes from the S vacancies in MoS_2_.

In 2021, Di Wu et al. again exploited the ultra-broadband absorption capability of PdSe_2_ and successfully developed a PdSe_2_/CdTe mixed van der Waals heterojunction [82]. Ultra-wide long-wave infrared detection at 10.6μm can be achieved at room temperature, as shown in Figure 4g,h. In 2022, Xiwei Zhang et al. constructed a high-quality van der Waals heterojunction by the in situ growth of WSe_2_ on n-Si and realized wide-spectrum detection from 200 nm to 1550 nm [86]. Huier Guo et al. in situ pulsed laser deposited β-In_2_Se_3_ on p-Si, and the heterojunction realized a wide spectral detection from 265 nm to 1300 nm [87]. In summary, the 2D/3D hybrid van der Waals heterojunction structure can provide a solution for preparing broadband detectors.

### 2.2. High-Responsivity

Responsivity (R) means the output signal per unit of incident light power. It can provide key information on the sensitivity of photodetectors. The responsivity of photodetectors is given by [17]:

(3) $$R=\frac{I_{ph}}{P_{in}}=\frac{e\eta_{e}}{h\upsilon }$$

where $I_{ph}$ is the photocurrent of the detector, and $P_{in}$ is the incident power of illumination light. The responsivity can be easily measured, so it is often used to evaluate other performances of the photodetectors, such as response spectrum, gain, bandwidth, linearity, and saturation level.

Detectivity is a direct performance evaluation index for IR photodetectors. It represents the signal to noise ratio divided by incident irradiance power. In order to eliminate the effects of the photo sensitive area, Jones proposed specific detectivity (D*) to specify detector performance. The quantum efficacy and background limited D* for photodiodes can be written as follows [17]:

(4) $$D^{*}=\frac{R}{i_{n}}\sqrt{A_{d}\Delta f}$$

where R is the responsivity, $i_{n}$ is the noise current spectra at 1 Hz bandwidth, $A_{d}$ is the area of the device, and Δf is the bandwidth. According to the present situation, 2D materials face the problem of low light absorption, leading to low quantum efficiency, especially in the infrared range. Monolayer graphene has the largest absorption coefficient among infrared-sensitive 2D materials [88]. Despite this, its optical absorption is only around 2%, meaning its EQE will be no more than 2% without gain. Therefore, photodetectors based only on 2D materials often have low quantum efficiency and responsivity [89]. 

**Figure 5:**
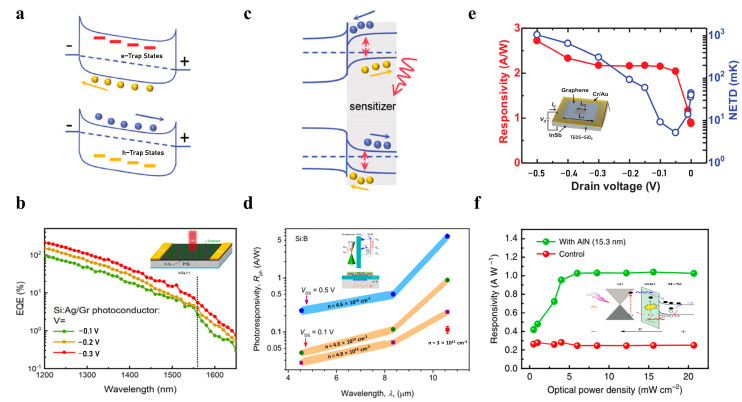
High-responsivity infrared photodetection. (**a**,**c**) Schematic diagrams of photogating effect [90] (copyright 2017, John Wiley and Sons). (**b**) Schematic diagram of graphene/Si:Ag heterostructure infrared detector and its EQE [92] (copyright 2022, Elsevier). (**d**) Schematic diagram of energy band structure and responsivity of graphene/Si:B detector [93] (copyright 2022, American Chemical Society). (**e**) Schematic diagram of graphene/undoped InSb heterostructure infrared detector and its responsivity [94] (copyright 2022, AIP Publishing). (**f**) Schematic diagram of graphene/insulation/silicon detector and its optical power-dependent responsivity [99] (copyright 2021, Springer Nature).

Fortunately, the photogating effect provides a solution to improve the responsivity of the detector. The photogenerated carriers are trapped in the impurity centers of the interface between the 2D materials and the dielectric layer or other materials. Then the trapped carriers will generate an effective electric field to regulate the channel property [90]. Usually, the processes of capture and release will last as long as milliseconds to seconds. As a consequence, the gain will be huge. As shown in Figure 5a, hole traps exist in n-type semiconductors. Electron–hole pairs are generated when illuminated [90]. The traps in the valence band capture the holes, and the probability of recombination of electrons and holes is significantly reduced, which prolongs the carrier lifetime and increases the gain.

Graphene and Si heterojunctions have been demonstrated to exhibit high responsivity and high external quantum efficiency in the visible and near-infrared regions [91]. In Figure 5b, Zijing Wang et al. used the photogating effect and high carrier mobility of graphene to solve the low responsivity of an Si:Ag device [92]. The low responsivity of the Si:Ag device was due to high doping, which carries a large number of thermal defects and amorphous components. Graphene is a carrier transport layer in the Si:Ag/Gr detector. Under the light, the electrons transit from the valence band to the deep-level defect states and then to the conduction band. By adjusting the gate voltage, the electrons and holes are effectively separated, and the electrons enter the graphene layer. With the excellent carrier migration ability of graphene, the quantum efficiency of the Si:Ag/Gr detector can reach 43.22% at 1310 nm compared with the quantum efficiency of the Si:Ag detector at 1310 nm, which is only 15.36%; the quantum efficiency has been greatly improved [92].

For the hybrid structure, the accumulation of carriers through the heterojunction interface will also lead to the photogating effect, as shown in Figure 5c. In 2022, Vinh X. Ho et al. used the photoionization of shallow impurities in highly doped Si:B substrates and nanostructures (microcavity and metallic plasma) on top of a graphene field effect transistor (FET) to achieve a high response in the MIRW [93]. As shown in Figure 5d, double photogating effects are utilized in the graphene/Si:B device structure. Due to the different work function of Si:B (5.07 eV) and graphene (4.56 eV), the energy band bends downward to form an electron potential well at the Si/SiO_2_ interface. Shallow impurities are photoionized under mid-infrared light. Holes remain in the valence band. Furthermore, photogenerated electrons are trapped in the acceptor states and attracted to the potential well at the Si/SiO_2_ interface, inducing extra holes in graphene channels through capacitive coupling. The responsivity of the graphene/Si:B detector in the mid-infrared can reach 5 A/W.

Low-doped 2D materials are challenging to obtain, but 2D materials combined with low-doped 3D materials bring a broad depletion region, reducing dark current and enhancing the photogating effect. As shown in Figure 5e, undoped InSb with a low carrier density is used to construct a graphene/InSb structure, increasing the width of the depletion layer at the interface. By inhibiting the diffusion of carriers, the dark current is significantly reduced to the magnitude of the nA level. In addition, it increases the junction capacitance and amplifies the carrier density modulation capability of graphene [94]. The responsivity exceeding 2 A/W in the mid-infrared is finally realized through the graphene/InSb heterojunction.

At the same time, to avoid the leakage current induced by surface state defects at the graphene/semiconductor interface [95], the introduction of a passivation layer [96,97] and nanoparticles/quantum dots for tunnel structures are usually used to suppress dark current and improve responsivity [98]. As shown in Figure 5f, Jun Yin et al. introduced 15 nm AlN as a wide-bandgap insulating layer between the graphene/n-Si interface to enhance the photoresponse [99]. Compared with other graphene/semiconductor devices, the dominant factor of the enhanced optical gain of the p–i–n device introduces an interfacial insulating layer. It acts as a barrier to block hole transport in Si, reducing dark current. At the same time, the electric field is concentrated in the high-resistance region, and the O/N defects in the AlN film will facilitate the tunneling and impact ionization of carriers, thereby enhancing the broad-spectrum response.

### 2.3. Fast-Response

Bandwidth (Δ*f*) is used to describe the response speed of photodetectors, an essential index for large-scale infrared focal plane detectors and communication receivers. The bandwidth of photodetectors can be roughly written as follows [17]:

(5) $$\Delta f=\frac{1}{2\pi \tau }$$

where τ is the response time of the photodetectors. 

The bandwidth, i.e., the response time, has many connected factors, such as the time of the photoelectric process carrier (τ_*pe*_), transit time (τ_*tr*_), RC time constant (τ_*RC*_), diffusion time (τ_*diff*_), and trapping effect (τ_*trap*_). The total response time can be written as follows [17]:

(6) $$\frac{1}{\tau }=\frac{1}{\tau_{pe}}+\frac{1}{\tau_{tr}}+\frac{1}{\tau_{RC}}+\frac{1}{\tau_{diff}}+\frac{1}{\tau_{trap}}$$

For 2D materials, the trapping effect is particularly striking because of the immature material growth and interface processing. Thus, most 2D infrared photoconductors face the trouble of long response time compared with thin film infrared photoconductors. The bandwidth advantage will be evident as the quality of the 2D materials improves due to the ultra-thin transport distance in the longitudinal dimension. However, often, the in-plane transport for the device with planar construction still takes much time. In addition, the RC time constant and diffusion time should be considered in junction devices [100]. Finally, the shortest time is the photoelectric process, but it is difficult to achieve. In a word, 2D materials could be a potential candidate for high-speed infrared photodetectors for scale advantages. However, the bandwidth of 2D photodetectors is still strongly limited by material quality [24,101].

At the beginning of the development of 2D photodetectors, the photoconductive gain was remarkable, but bandwidth was always on the back burner. However, infrared photodetectors with high bandwidth are very required for communications, high-speed imaging, and automatic target recognition [102]. Intrinsic 2D material has an ultrathin absorber [103]. Traditional bulk or thin film material photodetectors always utilize the thinning absorber layer to achieve high bandwidth [104]. However, most fabricated 2D material infrared photodetectors did not take advantage of the transport in the vertical directions. Therefore, the in-plane transmission of photogenerated carriers restricts the bandwidth. To expand the bandwidth, structure design becomes primary. It is expected to achieve high-bandwidth detection, efficient and fast light absorption and carrier separation, and fast response by optimizing the van der Waals heterojunction constructed of 2D and 3D materials.

**Figure 6:**
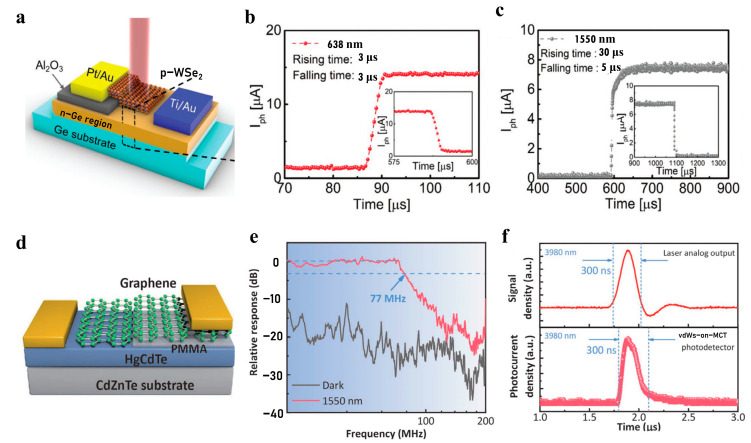
Fast-response infrared photodetection. (**a**) Optical microscope image of WSe_2_/Ge infrared detector [105] (copyright 2021, John Wiley and Sons). Its rising and falling time under light modulation at (**b**) 639 nm and (**c**) 1550 nm [105] (copyright 2021, John Wiley and Sons). (**d**) Schematic diagram of graphene/HgCdTe(MCT) van der Waals heterojunction photodetector [106] (copyright 2021, John Wiley and Sons). (**e**) Relative response versus switching frequency [106] (copyright 2021, John Wiley and Sons). (**f**) Time response of graphene/HgCdTe(MCT) photodetector under 3980 nm pulse laser [106] (copyright 2021, John Wiley and Sons).

In 2022, Chan Ho Lee et al. constructed a p-WSe_2_ and lightly doped n-Ge van der Waals heterojunction to achieve a fast response [105]. Figure 6a is an optical microscope image of the device. From Figure 6b,c, we can see that the photocurrent rise time and fall time are 3 μs in the light modulation cycle under 638 nm visible light, achieving a fast response. However, under 1550 nm infrared light, the photocurrent rise time (30 μs, comparable to other detectors based only on the van der Waals heterojunction) is longer than that under visible light. This is because under reverse bias and visible light irradiation, the photogenerated carriers generated in the p-WSe_2_ region can smoothly drift through the junction region and are affected little by the p-WSe_2_ and n-Ge junction regions, and a large photocurrent can be generated rapidly. However, when infrared light is irradiated, only the n-Ge region can generate photogenerated carriers, and the accumulated holes in the potential barrier cause the width of the depletion layer in the n-Ge region to decrease. Then the drift distance is short, and the response speed is low.

The 2D/3D hybrid van der Waals heterojunction also realizes fast detection in the infrared. Traditional HgCdTe (mercury–cadmium–telluride, MCT) photoconductive detectors have a large dark current, and the noise increases accordingly. It is difficult to achieve uncooled, high-performance infrared detection. Traditional MCT photovoltaic detectors introduce a large number of defects due to ion implantation, which increases dark current and response time. Yang Wang et al. innovatively complemented the advantages of graphene and MCT. MCT shows the highest absorption rate to middle and long-wave infrared currently, which makes up for the shortcomings of the weak light absorption of graphene. At the same time, graphene has high carrier mobility. Graphene was transferred onto MCTs by dry transfer to fabricate a high-quality, wrinkle-free van der Waals heterojunction. As shown in Figure 6d, the graphene/HgCdTe photodetector breaks through the shortcoming of the slow response of the existing uncooled long-wave infrared detectors and realizes fast response and high responsivity under black body radiation [106]. Response times are an order of magnitude faster than commercial room temperature MCT heterojunction detectors. The responsivity is as high as 2.5 A/W, and the quantum efficiency is as high as 85%. It can be seen from Figure 6e that the 3 dB bandwidth of the detector is as high as 77 MHz, and the response time is about 13 ns. Furthermore, as shown in Figure 6f, the response time of this detector can keep up with the fixed frequency of the mid-infrared pulsed laser. 

## 3. New Functional Device

### 3.1. Two-Color Infrared Detector

With the development of infrared detection technology, there is an urgent need for multi-band (multi-color) photodetectors [107]. The two-color photodetector can provide information in two bands, suppress the complex background of the target, recognize more spectral information than the single-color detector, and has better recognition performance in complex environments [48,108]. For example, the temperature difference between a missile surface and the tail flame is huge, but the two-color infrared detector can lock the missile surface and the missile tail flame simultaneously to eliminate interfering objects. Two-color detectors have essential application value in national defense, aerospace, remote sensing, and other fields. 

Two-color infrared detectors based on traditional 3D materials such as HgCdTe(MCT) generally need to use complex low-temperature cooling systems to reduce dark current [109], which does not meet the requirements of miniaturization. Furthermore, the junction interface will face problems such as lattice mismatch, which will affect the key technical indicators of the two-color detector, such as crosstalk and response. The preparation of hybrid structures based on 2D and 3D materials offers unique opportunities for developing two-color detectors. First, the thickness of the 2D/3D structure is enough to thoroughly absorb light and reduce crosstalk. Second, high-quality 2D/3D van der Waals heterojunction interfaces can be realized [110], enabling the excellent design of the two-color band structure. More importantly, the problem of lattice mismatch is inexistent in layered 2D materials, and the dark current generated by surface defects and thermal ionization will be significantly reduced [51,111].

**Figure 7:**
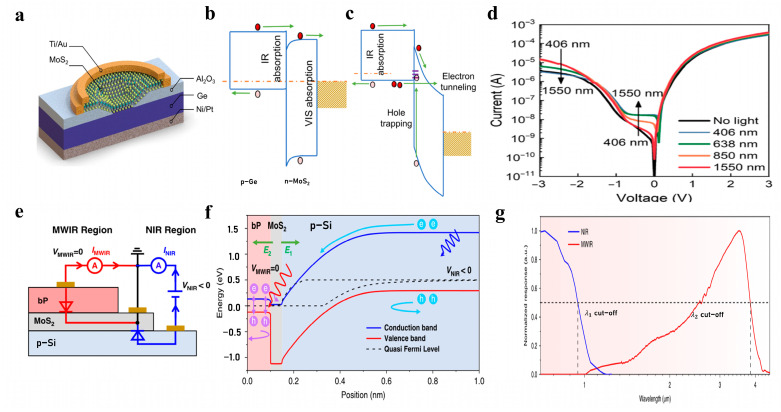
Two-color infrared detector. (**a**) Schematic diagram of p-Ge/n-MoS_2_ two-color infrared detector for VIS and infrared selective detection [112] (copyright 2021, American Association for the Advancement of Science). (**b**,**c**) Energy band diagrams under infrared light and visible light, respectively [112] (copyright 2021, American Association for the Advancement of Science). (**d**) Current–voltage curves under 1550 nm infrared light and 406 nm visible light [112] (copyright 2021, American Association for the Advancement of Science). (**e**) Schematic diagram of BP/MoS_2_/Si vdW two-color infrared detector for NWIR and MWIR detection [48] (copyright 2022, Springer Nature). (**f**) Energy band diagram of BP/MoS_2_/Si heterostructure [48] (copyright 2022, Springer Nature). (**g**) Normalized response of two-color detector at NIR and MWIR [48] (copyright 2022, Springer Nature).

As shown in Figure 7a, Aujin Hwang et al. developed a two-color detector based on a Ge/MoS_2_ van der Waals heterojunction with a near-photovoltaic/photoconductive mode for near-infrared (under low bias voltage) and visible light detection (high bias voltage), respectively [112]. As seen in Figure 7b, under small bias voltage, the energy band of MoS_2_ bends downward. The holes generated by absorbing visible light are blocked and recombined with electrons, resulting in limited carrier transport ability under visible light. The generated photo-generated carriers can pass through the heterojunction smoothly and reach the electrode, so near-infrared detection can be realized at this time. As seen in Figure 7c, the energy band structure of n-MoS_2_ is changed under a large bias voltage. The transport ability of photogenerated carriers in p-Ge due to near-infrared absorption is weakened. As shown in Figure 7d, compared with visible light, the photocurrent under near-infrared irradiation is very small. However, the photogenerated holes generated by visible light in the n-MoS_2_ region increase the tunneling current so that visible light detection can be realized under a large bias voltage.

In addition to satisfying dual-band detection, low crosstalk is also an important performance index for dual-color detectors. As shown in Figure 7e, Peisong Wu et al. designed a vertically stacked BP/MoS_2_/Si van der Waals heterostructure for two-color photodetection with near-infrared/mid-infrared blackbody sensitivity in a spatiotemporal coexistence mode [48]. Figure 7g shows this two-color infrared photodetector with ultra-low crosstalk of about 0.05% at room temperature. As shown in Figure 7f, by constructing a PNP junction with a back-to-back structure, two built-in electric fields with opposite directions were successfully introduced. The separation and independent detection of photogenerated carriers in two bands were realized [48]. Under reverse bias, the photogenerated carriers generated by near-wavelength infrared (NWIR) light radiation are separated, electrons are pushed to MoS_2_ by the built-in electric field E1, and holes are received by the P-Si electrode. Under zero bias, the photogenerated carriers generated by mid-wavelength infrared (MWIR) light radiation are separated by the built-in electric field, and the electrons are pushed to the MoS_2_ end by the built-in electric field E2 and collected by the electrode. At the same time, the holes are received by the BP electrode [48].

### 3.2. Polarization Infrared Detector

Photodetectors generally only detect light intensity information. However, a single light intensity information cannot meet the actual detection needs in complex application scenarios with strong interference backgrounds, such as air-to-ground detection and optical stealth. Taking advantage of different polarization states with light signals reflected by objects of different materials and shapes, the polarization detector can enhance the detection dimension of the target by simultaneously detecting the polarization and light intensity information, thereby upgrading the detection and recognition capabilities [113], specifically, object shape, roughness, and medium properties. Traditional polarization detectors usually need to use optical polarization elements such as metal gratings to realize the detection of polarized light [114]. It increases the structural complexity of the detectors and the complexity of the systems and limits the imaging resolution. The new generation of polarization detection systems will develop in the direction of miniaturization, integration, and flexibility. Therefore, it is necessary to find new materials and device structures for realizing a new generation of polarization detectors with high polarization sensitivity and high resolution. The emergence of van der Waals layered materials is of great help in realizing the miniaturization and flexibility of polarization detectors [115,116]. The unique asymmetric lattice structure of some 2D materials endows them with planar anisotropic electrical and optical properties [117]. It can replace the function of optical polarization elements in traditional polarization detectors, thereby realizing the miniaturization of polarization detection.

**Figure 8:**
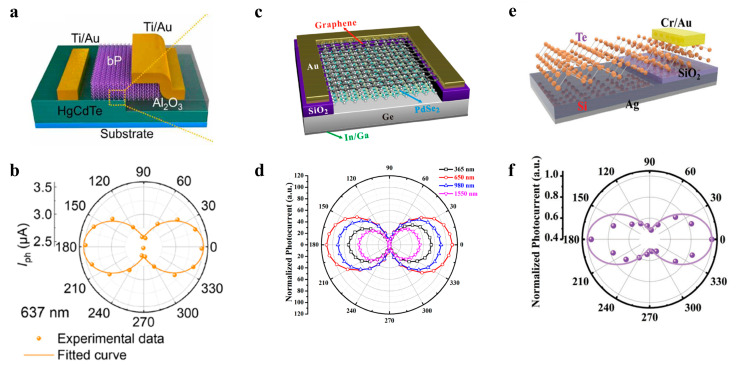
Polarization infrared detector. (**a**) Schematic diagram of polarization-sensitive HgCdTe/BP detector [118] (copyright 2021, American Association for the Advancement of Science). (**b**) Photocurrent experiment data with 637 nm laser illumination of HgCdTe/BP detector [118] (copyright 2021, American Association for the Advancement of Science). (**c**) Schematic diagram of Gr/PdSe_2_/Ge heterojunction photodetector with highly polarization-sensitive [119] (copyright 2019, American Chemical Society). (**d**) Photocurrent of Gr/PdSe_2_/Ge detector as a function of polarization angle at zero bias [119] (copyright 2019, American Chemical Society). (**e**) Schematic diagram of Te/Si heterojunction detector [120] (copyright 2022, Royal Society of Chemistry). (**f**) Normalized photocurrent with 635 nm laser at zero bias voltage [120] (copyright 2022, Royal Society of Chemistry).

BP shows high carrier mobility, changeable bandgap with layer number, and anisotropic crystal structure, widely used in developing polarization detectors. Due to the weak light absorption ability of 2D materials, the construction of 2D/3D hybrid van der Waals heterojunction shows excellent potential. As shown in Figure 8a,b, Hanxue Jiao et al. proposed a HgCdTe/BP hybrid heterojunction, which realized polarization-sensitive detection from the visible to the mid-wave infrared [118]. At the same time, the device exhibited excellent detection performance. The broken-gap band alignment heterojunction light-to-tunneling brings a large zero-polarized photocurrent and low dark current.

In 2019, as shown in Figure 8c, Di Wu et al. prepared a graphene/PdSe_2_/Ge heterojunction polarization detector with a polarization sensitivity as high as 112.2 [119]. The main reasons for the excellent performance were as follows: Ge has a large absorption coefficient; graphene as a transparent electrode improves the carrier collection efficiency and inhibits the recombination of carriers; PdSe_2_ possesses a low-symmetry crystal structure with broken inversion symmetry and exhibits prominent anisotropy. Moreover, the high-quality van der Waals vertical junction enhances the polarization response of PdSe_2_. As shown in Figure 8d, at 650 nm (52.6 mW/cm^2^), the polarization sensitivity is as high as 112.2. In 2021, Di Wu et al. again exploited the excellent anisotropy of PdSe_2_ and successfully developed a PdSe_2_/CdTe mixed van der Waals heterojunction [82]. The PdSe_2_/CdTe heterojunction detector also demonstrates high sensitivity to polarized infrared light, with a polarization sensitivity of 4.4. 

Besides for BP and PdSe_2_, Te has also been used in the development of polarization detectors due to its structural asymmetry, excellent binomial color ratio, and high air stability (42 months). As shown in Figure 8e, Tao Zheng et al. prepared a Te/Si mixed van der Waals heterojunction, which has a large built-in electric field to quickly separate widely-generated carriers and exhibit excellent anisotropy [120]. Figure 8f shows photocurrents at different polarization angles, and the photocurrent anisotropy ratio is 2.1 under 635 nm illumination.

## 4. Summary and Outlook

This paper reviews 2D/3D hybrid van der Waals heterojunction detectors, which provide new solutions for the performance improvement of current detectors (including broadband, high responsivity, and fast response) and the development of new functional devices (including two-color detectors and polarization detectors). However, there are still challenges to be further explored. In the following, we will elaborate on three aspects: materials, device, and system.

For materials, we hope to obtain nearly wrinkle-free and residue-free 2D/3D heterojunctions. The existing methods for forming 2D/3D heterojunctions are mainly divided into top-down and bottom-down methods. Top-down mainly refers to the use of mechanical stripping to transfer materials to 3D semiconductor substrates. However, mechanical stripping will make the uniformity of materials uncontrollable, and the crystal size is limited [121]. Moreover, regardless of dry transfer or wet transfer, there is no guarantee that there will be no residue, no damage, and absolute cleanliness [122]. Bottom-down mainly includes chemical vapor deposition (CVD) [123], molecular beam epitaxy (MBE) [124], atomic layer deposition (ALD) [125,126], electron beam epitaxy (EBE), etc. [127,128], which are suitable for large-scale wafer-level growth. However, they are expensive, and the conditions are harsh, not suitable for all 2D materials. Therefore, we urgently need to find a controllable, large-area, clean, non-destructive, and scalable technology to achieve a high-quality and high-precision 2D/3D detector. In addition, the selection of appropriate 2D materials and 3D materials according to the characteristic is important. Compared with 3D materials, the light absorption of 2D materials is relatively weak. Therefore, for most 2D/3D photodetectors, 2D materials are mainly used as carrier transport layers or junction layers to improve the bandwidth or detectivity. Specially, some new functional devices can also be fabricated using the particular characteristics of 2D materials, such as polarization detectors and multicolor detectors. Table 1 recommends the 2D materials and 3D materials required for the preparation of 2D/3D infrared detectors with different performance requirements. For the improvement of detector performance, narrow bandgap 2D materials are used, such as PdSe_2_, PtSe_2_, etc., and 3D materials with strong light-absorption capabilities, such as Si, Ge, etc., to achieve broadband; 3D materials with minority carriers featured long lifetime are used, such as InSb(77 K), HgCdTe(77 K), combined with 2D materials with photogating to achieve high-responsivity; 2D materials with high carrier mobility as carrier transport layers are used, combined with 3D materials with strong light-absorption to achieve fast response. For the preparation of new functional devices, two-color detectors can be realized through ingenious and appropriate energy band design; polarization detectors can be realized by using anisotropic two-dimensional materials, such as BP, PdSe_2_, ReSe_2_, ReS_2_, etc., and strong light-absorbing 3D materials. For devices, there are still many new areas of research on the physical mechanism and band structure design of 2D/3D heterojunctions to be discovered. It is still necessary to explore the possibility and adaptability of more combinations of different 2D and 3D materials. Most of the 2D/3D detectors reported so far are artificially prepared, which is uncertain and not suitable for mass production and industrialization. We need mature 2D/3D device preparation technology to improve environmental stability. For the system, most of the current 2D/3D detectors are unit devices, which have not yet met the requirements of integration, large area, array, and commercial production. Therefore, we believe that with the strict development of material technology, device physics, and systems, 2D/3D hybrid van der Waals heterojunction detectors are expected to make great achievements in the field of infrared detection.

## Figures and Tables

**Table 1:** Material selection for 2D/3D infrared photodetectors.

2D/3D Infrared Detectors		3D Materials	2D Materials
Performance improvement	Broadband	Strong absorption: Si, Ge, InGaAs, HgCdTe, InAsSb, InSb	Narrow bandgap: PdSe_2_, PtSe_2_
	High-responsivity	Minority carriers with long lifespan: InSb(77 K), HgCdTe(77 K)	Photogating: graphene, MoS_2_, In_2_Se_3_
	Fast-response	Strong absorption: Si, Ge, InGaAs, HgCdTe, InAsSb, InSb	High carrier mobility: graphene, BP
New functional device	Two-color infrared detector	Ingenious and reasonable energy band design	
	Polarization infrared detector	Strong absorption: Si, Ge, InGaAs, HgCdTe, InAsSb, InSb	Anisotropy: BP, PdSe_2_, ReSe_2_, ReS_2_
